# Quantitative comparison of the neutralizing capacity, immunogenicity and cross-reactivity of anti-TNF-α biologicals and an Infliximab-biosimilar

**DOI:** 10.1371/journal.pone.0208922

**Published:** 2018-12-11

**Authors:** D. J. Buurman, T. Blokzijl, E. A. M. Festen, B. T. Pham, K. N. Faber, E. Brouwer, G. Dijkstra

**Affiliations:** 1 Department of Gastroenterology and Hepatology, University Medical Center Groningen, University of Groningen, Groningen, The Netherlands; 2 University of Groningen, University Medical Center Groningen, Department of Laboratory Medicine, Groningen, The Netherlands; 3 Department of Rheumatology and Clinical Immunology, University Medical Center Groningen, University of Groningen, Groningen, The Netherlands; Boston University Henry M Goldman School of Dental Medicine, UNITED STATES

## Abstract

**Introduction:**

TNF-α-neutralizing antibodies, such as infliximab (IFX) and adalimumab (ADA), are effective in the treatment of inflammatory bowel diseases (IBD), but they are expensive and become ineffective when patients develop anti-IFX or anti-ADA antibodies (ATI and ATA, respectively). Second-generation anti-TNF-α antibodies, such as Golimumab, Etanercept, Certolizumab-pegol and IFX biosimilars, may solve these issues.

**Aim:**

To determine the neutralizing capacity of first- and second generation anti-TNF-α antibodies and to determine whether ATI show cross-reactivity with the IFX biosimilar CT-P13 (Inflectra).

**Methods:**

TNF-α neutralization was measured using a quantitative TNF-α sensor assay consisting of HeLa 8D8 cells that express the Green Fluorescence Protein (GFP) under control of a NF-кB response element. All available anti-TNF-α drugs and the IFX biosimilar CT-P13 (Inflectra) were tested for their TNF-α-neutralizing capacity. In addition, patient sera with ATI were tested for their potential to block the activity of IFX, IFX (F)ab^2^-fragment, biosimilar CT-P13 (Inflectra) and ADA.

**Results:**

TNF-α strongly induced GFP expression in Hela 8D8 cells. Higher concentrations of first-generation anti-TNF-α drugs were required to neutralize TNF-α compared to the second-generation anti-TNF-α drugs. Serum of IBD patients with proven ATI blocked TNF-α-neutralizing properties of IFX biosimilar CT-P13 (Inflectra), whereas such sera did not block the effect of ADA.

**Conclusion:**

The second-generation anti-TNF-α drugs show increased TNF-α-neutralizing potential compared to first-generation variants. ATI show cross-reactivity toward IFX biosimilar CT-P13 (Inflectra), consequently patients with ATI are unlikely to benefit from treatment with this IFX biosimilar.

## Introduction

TNF-α blocking agents, such as infliximab (IFX) and adalimumab (ADA), are highly effective in the induction and maintenance of remission in Crohn’s disease (CD) and ulcerative colitis (UC) and have led to remarkable improvements in the therapy of CD and UC. [1–8] However, TNF-α blocking agents are very expensive and currently constitute the majority of the costs of IBD therapy.[9] IFX-biosimilars, such as CT-P13 (Inflectra/ Remsima),Celltrion) and Flixabi (Biogen) are available now.[10–13] These biosimilars are expected to have the same specificity and the same sequence as the original molecule IFX (Remicade), but are marketed at a much lower price than the first-generation anti-TNF-α blocking agents, which will make treatment of these diseases more cost-effective.

Besides the cost of these drugs, another problem in the treatment with TNF-α blocking agents is that approximately 10%-21% of the patients annually lose their response to the treatment partially due to formation of antibodies against the drug (antigenicity), causing low trough levels.[14–16] Antibodies against IFX (ATI) and ADA (ATA) are correlated with lower trough levels and reduce the efficacy of IFX therapy. [17–19]

Currently second generation TNF-α blocking agents as Certolizumab-PEGOL (Cimzia) and Golimumab are shown to be effective in the treatment of CD and UC. [20–22]

It is unclear whether ATI always neutralize the anti-TNF-α drug and whether they show cross-reactivity towards other available anti-TNF-α therapeutics or IFX biosimilar.

Here, we compared the TNF-α-neutralizing capacity of all commercially available anti-TNF-α drugs. Furthermore, we tested the neutralizing capacity of ATI, as well as their cross-reactivity with IFX, ATI, ADA and ATA. Finally, we tested if ATI towards the original IFX cross react with the biosimilar of infliximab (CT-P13 (Inflectra)).

## Material and methods

### Patients

In a retrospective cohort, 23 IFX (Remicade) treated IBD patients were identified with anti-IFX antibodies (ATI) and their clinical parameters were collected from their electronic patient dossier. This is a retrospective study on biobank material. This biobank was approved by the ethical review board of the UMCG approved August 5^th^, 2009, protocol number METc 2008.338. Written, informed consent was obtained from each patient included in the study. Within this informed consent patient agrees to link the patient’s biobank ID to the electronic patient’s dossier. The authors had full access to the electronic patient dossier. The study protocol conforms to the ethical guidelines of the 1975 Declaration of Helsinki as reflected in a prior approval by the institution’s human research committee.

### Neutralizing effect of anti-TNF-α agents

To investigate the difference between the neutralizing effect of IFX, IFX (F)ab^2^-fragment, ADA, Etanercept, Certolizumab and Golimumab, we used HeLa 8D8 cells that express the Green Fluorescence Protein (GFP) under control of the NF-kB response element [23].

Cultures of HeLa 8D8 cells were maintained in plates at a density of 250,000 cells/well in 1 mL Dulbecco’s-modified Eagle medium (DMEM), supplemented with 10% heat-inactivated fetal calf serum (FCS) and incubated in an atmosphere of 5% CO_2_ at 37°C. The peak of TNF-α (1 ng/ml)-induced GFP expression was observed after 16 h of incubation, which was chosen for all further experiments. Reagents were added directly to the cells after seeding them and incubated for 16 hours. After incubation, cells were suspended in 350 μl of ice-cold phosphate-buffered saline (PBS) supplemented with 1% bovine serum albumin (BSA) and kept on ice until analysis. The number of GFP-positive cells was quantified by flow cytometry using a FACSCalibur^™^ flow cytometer. The fluorescent signal was measured in at least 10,000 cells. Different concentrations of IFX, IFX biosimilar CT-P13 (Inflectra), ADA, Certolizumab, Etanercept and Golimumab (0-5-10-20-40-100 ng/mL) were analyzed for their neutralizing capacity on 1 ng/mL TNF-α. All experiments were conducted four times.

### Antibodies to Infliximab (ATI)

Blood samples of 23 IBD patients with ATI were collected. The concentrations of antibodies against IFX and the serum levels of free IFX were determined by Sanquin, Amsterdam, The Netherlands [24,25]. We evaluated the clinical consequences and the *in vitro* blocking effect of sera of patients with proven ATI. Serum samples that contained ATI were incubated with 1 ng/mL of TNF-α and 10–40 ng/mL of IFX were added to the Hela 8D8 cells. The quantity of serum was adjusted to a concentration of 7 AU/mL of ATI. Different amounts of normal human serum were used as negative controls.

### Cross-reactivity in antigenicity

Cross-reactivity between antibodies against IFX and ADA was tested using sera of 2 CD patients with ATI (7 AE/ml) and sera of 1 AS and 1 PsA patient with ATA (30 AE/mL).

Sera with ATI of 6 different IBD patients were used to show antigenic cross-reactivity between IFX and the IFX biosimilar CT-P13 (Inflectra).

### Statistical analysis

Continuous variables were given as mean and range and categorical data as numbers with percentages. Differences between groups, at baseline, different follow-up times, were evaluated by the Student–*t-* test and otherwise the Mann-Whitney U test, depending on normality of the data, for continuous data. All tests of significance were two-sided, with p-values of <0.05 assumed to indicate significance. Statistical analyses were performed using IBM SPSS Statistics version 25.0 (Armonk, NY, USA) unless otherwise mentioned.

## Results

### Patient characteristics

Blood samples of 23 IBD patients with ATI were used to test the blocking effect of the neutralizing effect of IFX. The characteristics of these patients are shown in Table 1.

**Table 1:** Characteristics of 23 IFX (Remicade) treated IBD patients with ATI.

Characteristics	Value
Gender	
Male	7
Female	16
Age (yr)	
Mean	43.9
Range	25–74
Disease	
Crohn’s disease (CD)	19
Ulcerative colitis (UC)	4
Localization disease- no of patients	
Colitis	10
Ileocolitis	9
Ileitis	4
Fistulas	6
Concomitant medication to infliximab	
Corticosteroids	8
Azathioprine/6-mercaptopurine/Methotrexate	15
Cyclosporine	1
No other medication	5
Dosage Infliximab, mean (range)	5.6 mg/kg (5-10mg/kg)
No of infusions Infliximab, mean (range)	8.9 (4–20)
Anti-IFX antibody concentration mean (range)	921.3 (12–7855) AU/ml
Consequences antibodies against IFX.	
Loss of response	14
Infusion reaction	5
Lack of response	4

### The different TNF-α neutralizing effect of the anti-TNF-α agents

**Fig 1:**
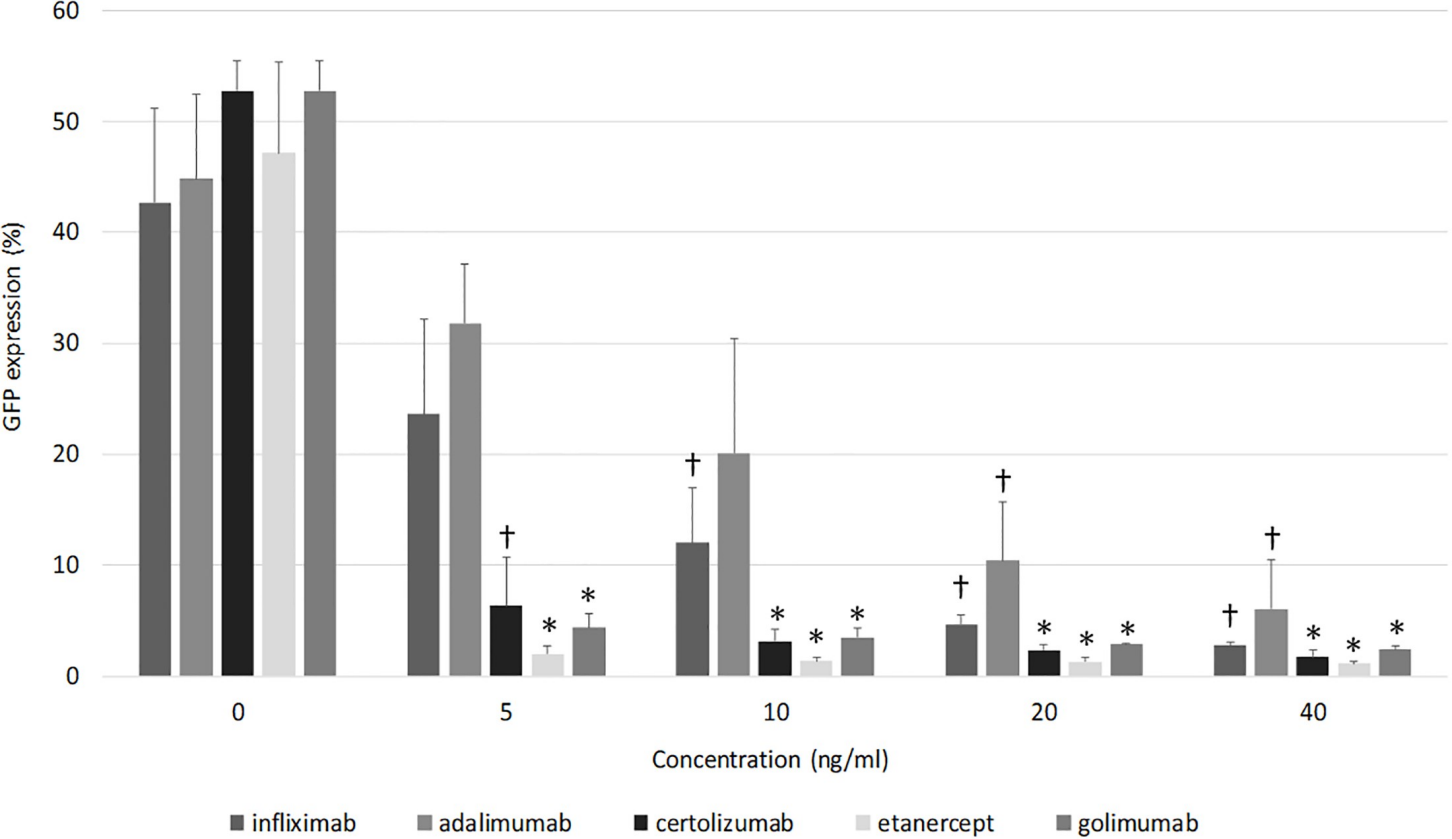
The TNF-α (1ng TNF-α) neutralizing effect of IFX, ADA, Certolizumab, Etanercept and Golimumab in different concentrations is illustrated. The higher the concentration of the biological, the stronger the neutralizing effect. † is p<0.05 as compared to 0ng/ml; * is P<0.001 as compared to 0ng/ml.

**Fig 2:**
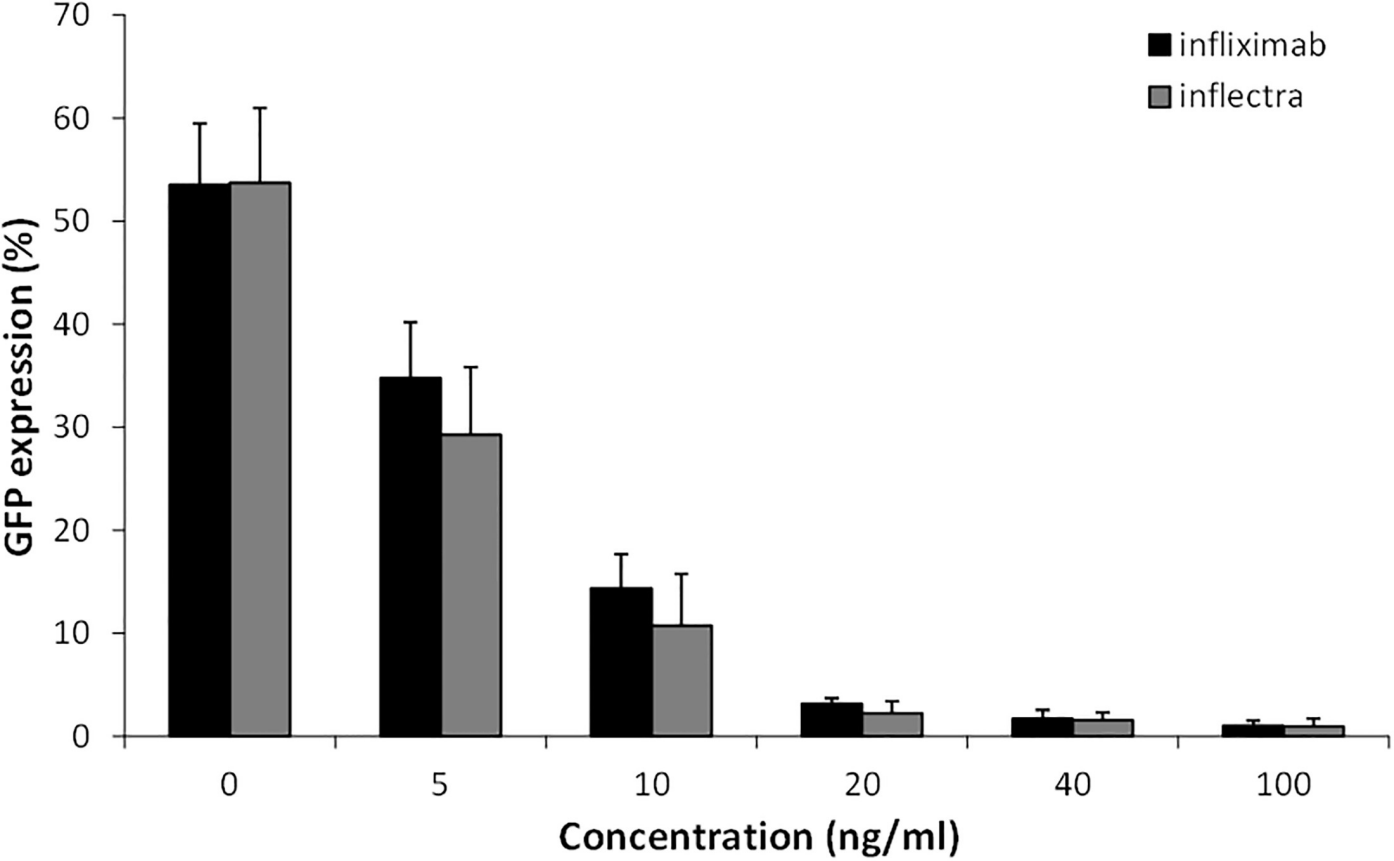
No difference in TNF-alfa neutralizing effect comparing IFX with IFX biosimilar CT-P13 (Inflectra).

A 16 h treatment with 1 ng/ml TNF-α resulted in a strong induction of GFP-positive HeLa 8D8 cells (approximately 50%), validating their use as TNF-α reporter cells (Fig 1). Next, IFX, ADA, Certolizumab, Etancercept and Golimumab were evaluated for their TNF-α neutralizing effect. The anti-TNF-α drugs were tested at 0-5-10-20-40-100 ng/mL in the presence of 1ng/ml TNF-α. All anti-TNF-α drugs dose-dependently reduced the number of TNF-α-induced GFP positive HeLa 8D8cells. Etanercept, Certolizumab and Golimumab demonstrated a stronger (p<0.001) neutralizing effect compared to IFX and ADA (Fig 1). There is no statistical difference in the neutralizing effect of the IFX biosimilar CT-P13 (Inflectra) compared to IFX (Fig 2). Because different batches of IFX exist we show that even 4 year old IFX behaved similar to the new batch of IFX that was primarily used in our study. We tested fresh and 4 year-old IFX from different batches in solubilized and dry conditions and found no statistical difference in the TNF-α neutralizing capacity. (S1 Fig).

### ATI block the TNF-α-neutralizing effect of IFX

Sera of 17 different IBD patients with different concentrations of ATI were added together with 1 ng/mL of TNF-α and 10–40 ng/mL of IFX to the HeLa 8D8 cells.

**Fig 3:**
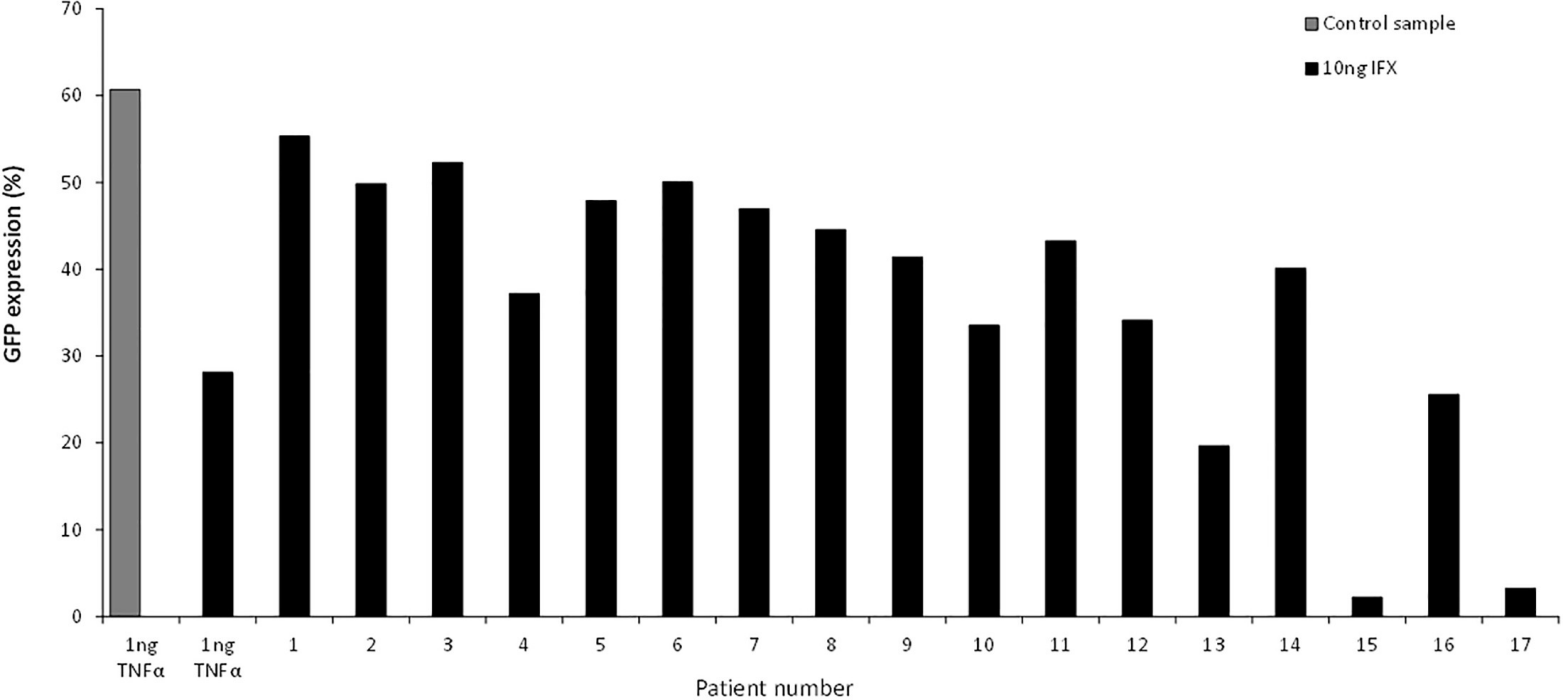
The blocking effect of sera of 17 IFX treated IBD patients with ATI. Patient 15 and 17 had low ATI, 46 AE/ml and 59 AE/ml respectively.

We descriptively show that there is a blocking effect of ATI of the 17 different IBD-patient’s serum samples on the neutralizing effect of IFX as shown in Fig 3.

Sera of patient 15 and 17 had a low blocking effect possible due to that they had lower levels of ATI, 46 AE/ml and 59 AE/ml, respectively. We show in the S2 Fig that a higher ATI concentration does have a stronger blocking effect of IFX and IFX biosimilar CT-P13 (Inflectra) (S2 Fig).

### The cross-reactivity between anti-TNF-α drugs and anti-TNF-α antibodies

**Fig 4:**
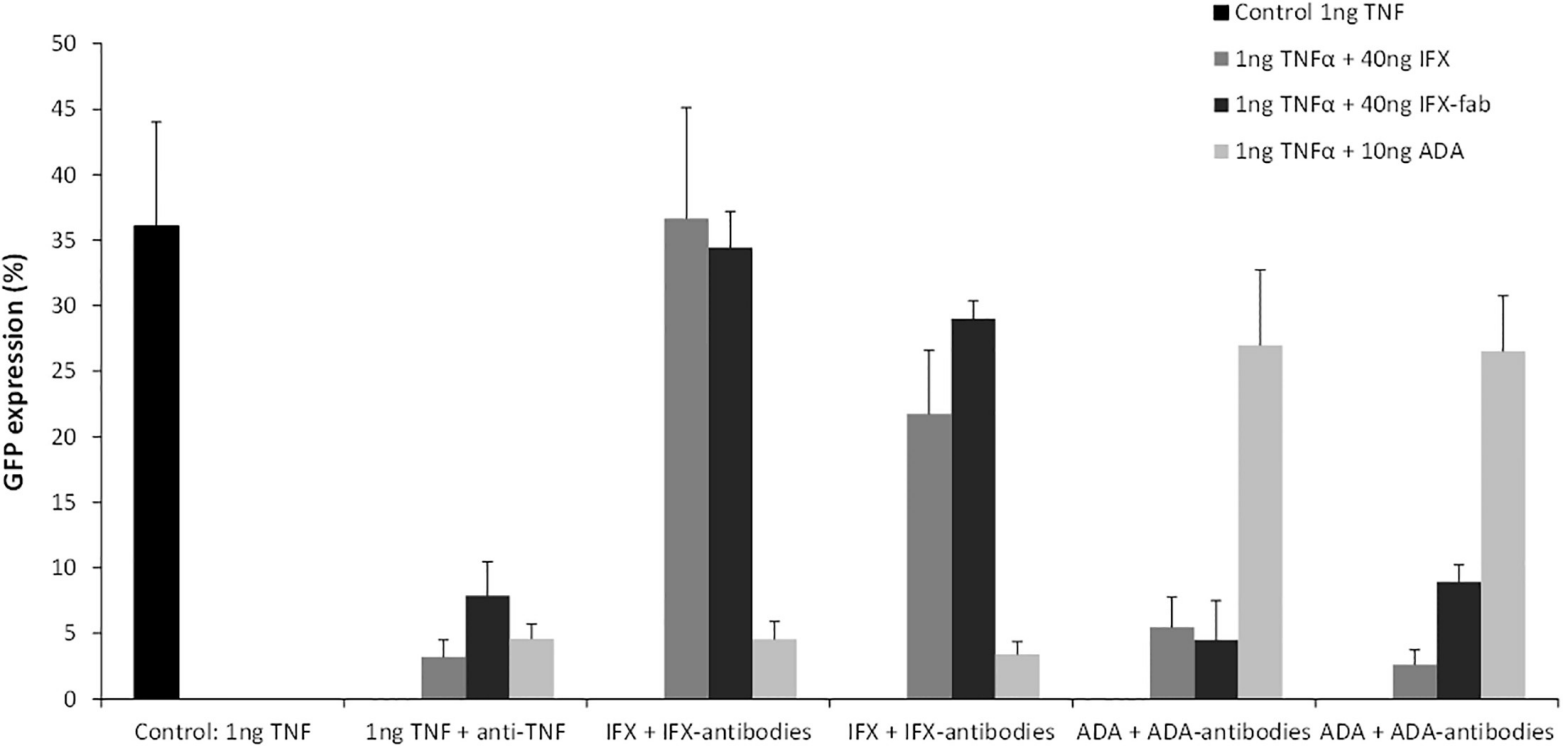
Blocking experiments with IFX, F(ab)2 of IFX and ADA in two samples of CD patients with ATI and 2 samples of RA patients with anti-ADA antibodies. There is no cross reactivity among ATI and anti-ADA and the IFX antibodies are directed against the F(ab)2-fragment of IFX.

**Fig 5:**
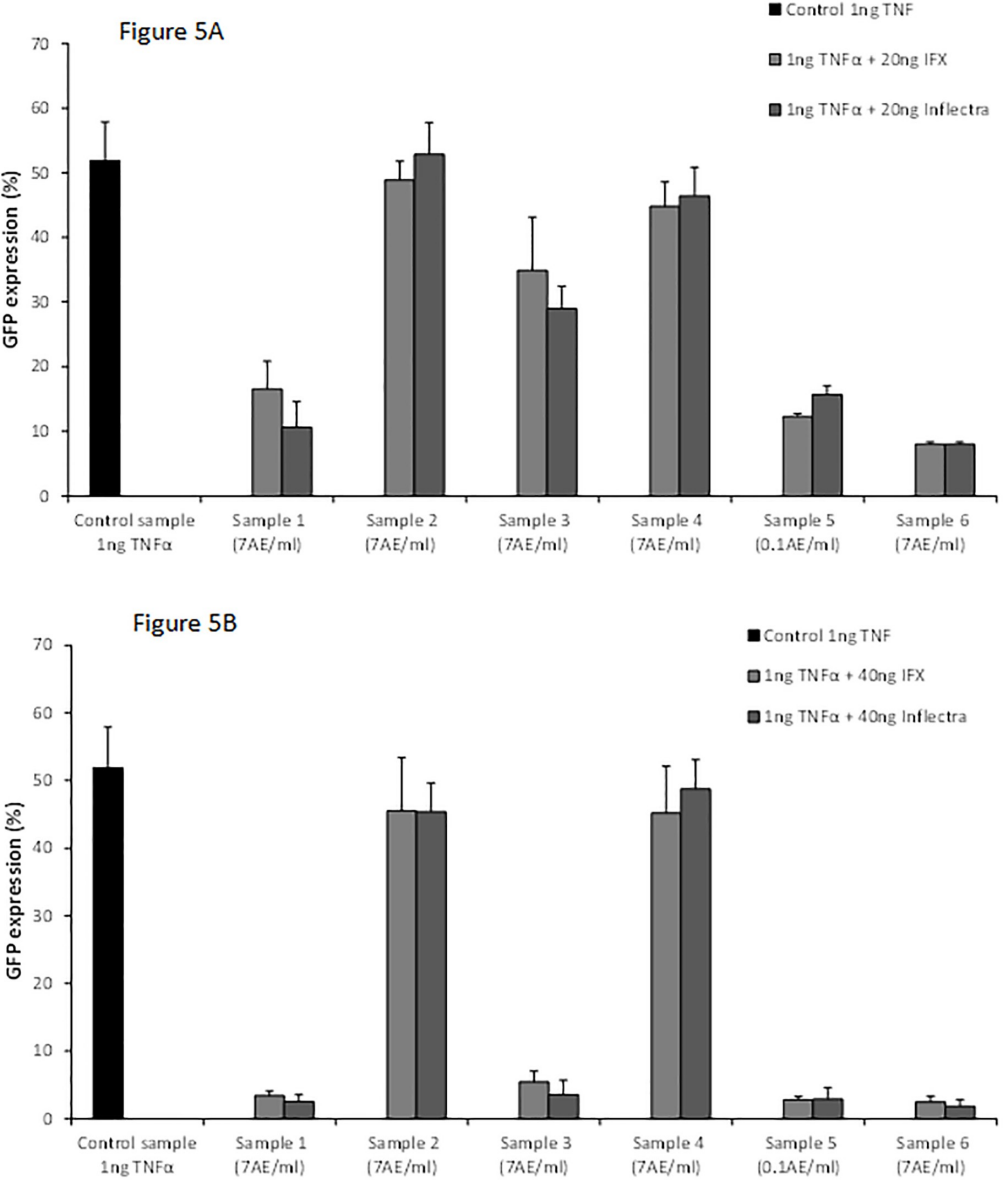
**A and B**. Blocking experiments with 20 (Fig 5A) and 40 (Fig 5B) ng IFX and its CT-P13 (Inflectra) biosimilar showing the cross reactivity of ATI with its biosimilar.

Serum samples of 2 IBD patients with ATI and serum samples of 2 RA patients with ATA were used to evaluate the cross-reactivity between ATI and ADA, and ATA and IFX, respectively. The sera with ATI inhibited the effect of the IFX F(ab)2-fragment, but did not block the neutralizing effect of ADA (Fig 4). Therefore, it is likely that the IFX antibodies are directed against the F(ab)2-fragment. Sera of patients with ATA did not block the neutralizing effect of IFX. Therefore, no antigenic cross-reactivity was found between IFX and ADA. In contrast to ADA, ATI shows a blocking effect of the neutralizing capacity of the IFX biosimilar CT-P13 (Inflectra) (Fig 5). This means that the IFX biosimilar CT-P13 (Inflectra) has the same immunogenic epitopes as IFX.

## Discussion

Using an *in vitro* reporter assay, we show in this study that Etanercept (ETA), Certolizumab and Golimumab are more effective in neutralizing TNF-α compared to the first generation anti-TNF-α drugs IFX and ADA. We also show that serum with proven ATI blocked the TNF-α-neutralizing properties of IFX, the F(ab) ^2^ of IFX, and the IFX biosimilar CT-P13 (Inflectra), whereas ATI did not block the effect of ADA.

IFX was the first anti-TNF-α biological for the treatment of IBD patients. The ACCENT1 trial showed that CD patients treated with IFX therapy were 3 times more likely to have clinical remission in the maintenance phase.[3] The second anti-TNF-α biological approved for the treatment of CD was ADA, which also showed significantly more induction of remission and maintenance remission rates compared to placebo.[7,26] ADA and IFX are considered first generation biologicals. The second generation biologicals, like certolizumab pegol and golimumab, have been approved for use in patients with respectively CD and UC.[20–22]. There are no publications yet that directly compare the neutralizing capacity of first and second generation anti-TNF-α agents. Patil *et al* reported in a retrospective review that there was no difference in clinical remission rates between IFX, ADA and Certolizumab pegol.[27]

Recently, biosimilars of IFX, such as CT-P13 (Remsima/Inflectra), manufactured by Celltrion and Flixabi manufactured by Biogen came available for the treatment of IBD.[12,13] The term biosimilar suggests that these new biologicals have the same properties as the original biological. The recent NOR-Switch study showed that a switch to biosimilar infliximab (CT-P13) from originator infliximab is not inferior to continued treatment with the originator and that patients can be safely switched. [28]

In this study we show that there is no difference in either the anti-TNF-α effect or antigenicity of IFX and its biosimilar CT-P13 (Inflectra) *in vitro*. The ATI seems to be directed against the F(ab)2-fragment of the anti-body and since an antibody reaction is very specific this proves similarity. These findings correspond to the findings with Remsima.[29] This is important because the costs of IBD treatment are mainly driven by first and second generation anti-TNF- α agents, and biosimilars are generally much cheaper.[9] As a result of the specificity of biosimilars, IBD patients without antibodies against Infliximab could in theory be treated with or switched to a cheaper biosimilar. In vitro study showed that the same holds for adalimumab biosimilar, but its clinical effect has to be awaited).[30] In accordance with clinical observations we show that there is no cross-reactivity between antibodies against IFX and ADA.

As shown in clinical studies, IFX-treated patients with loss of response due to the development with ATI can be switched safe and effective to ADA and vice versa.

As the expiration date for IFX was considered to be only 24 hours we tested fresh and 4 year-old IFX from different batches in solubilized and dry conditions and found no difference in the TNF-α neutralizing capacity. Therefore, spill of this expensive biological can probably be reduced by compounding and storing IFX in sterile and cool conditions, according to normal practice for antibody storage. Our study has some limitations. First, the sample size is relatively small and therefore data is restricted to observations and hypotheses and cannot reveal (causal) mechanisms. Any additional subgroup analysis have not been performed due to size of the study, therefore potential clinical implications as patient tailored therapy remains hypothetical. Strengths include our well-characterized cohort, and uniform collection of laboratory data.

In conclusion, we show that the second generation anti-TNF-α drugs show increased TNF-α neutralizing potential compared to first generation anti-TNF-α drugs. We also show that IFX and the IFX biosimilar CT-P13 (Inflectra) have the same neutralizing capacity as the original IFX. Furthermore, we show that ATI show cross-reactivity toward the IFX biosimilar CT-P13 (Inflectra) proving that the CT-P13 (Inflectra) IFX biosimilar also has the same antigenic properties and therefore the clinical implication is that patients with ATI should not be switched to IFX biosimilar. However, patients treated with IFX without IFX antibodies can be switched safely and effectively to this cheaper IFX biosimilar in order to reduce costs of anti-TNF-α treatment in IBD. Patients with ATI could benefit from a switch to adalimumab as there is no cross-reactivity.

## Supporting information

S1 Fig No difference in the neutralizing capacity of new and 4 year old IFX. (TIF)

S2 Fig Stronger blocking effect of the neutralizing capacity of IFX and CT-P13 (Inflectra) with increased concentrations of ATI. (TIF)

S1 File Data set. (XLSX)

## Abbreviations

ADA: Adalimumab
AS: Ankylosing spondylitis
ATA: Antibodies to Adalimumab
ATI: Antibodies to Infliximab
CD: Crohn’s disease
IBD: Inflammatory Bowel Diseases
IFX: Infliximab
PsA: Psoriatic Arthritis
Pt: Patient
RA: Rheumatoid Arthritis
TNF-α: Tumor Necrosis Factor alpha
UC: Ulcerative Colitis
