# Supplementary figures and images for: Quantitative comparison of the neutralizing capacity, immunogenicity and cross-reactivity of anti-TNF-α biologicals and an Infliximab-biosimilar
Source: PLoS One. 2018 Dec 11;13(12):e0208922. doi: 10.1371/journal.pone.0208922 (PMC6289430; doi:10.1371/journal.pone.0208922)

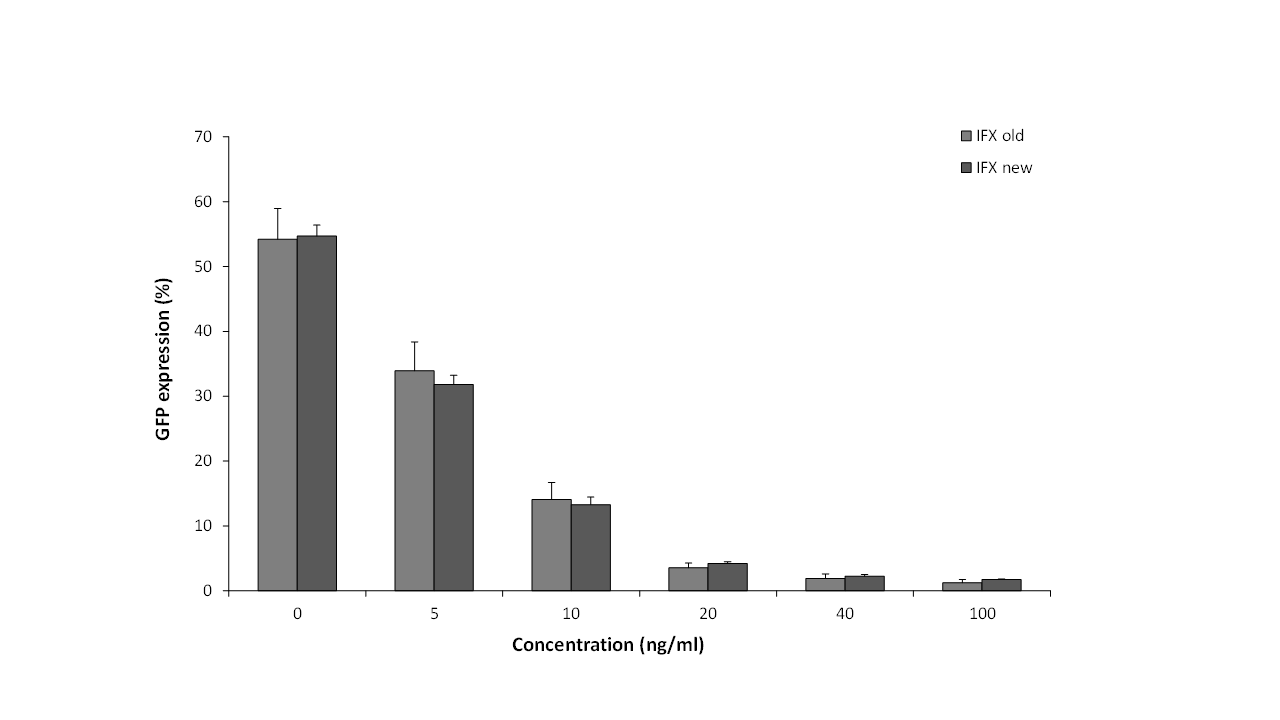

Supplement: S1 Fig — (TIF) [file pone.0208922.s001.tif]

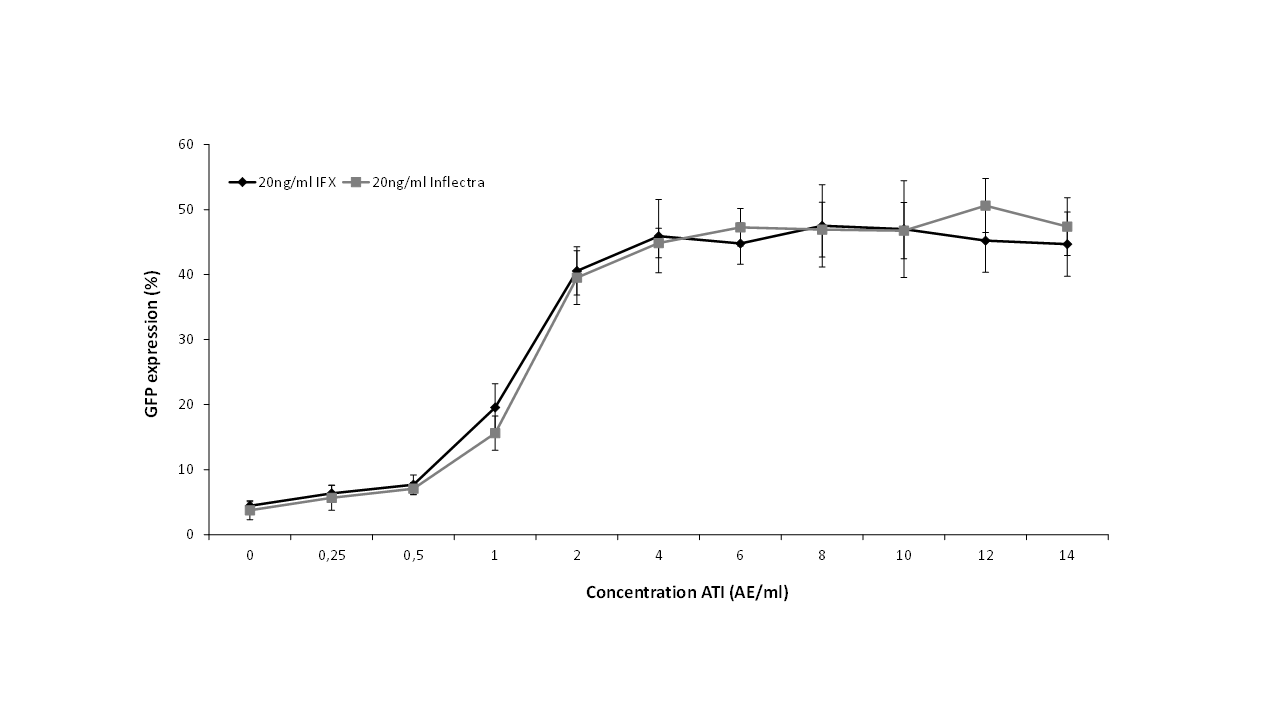

Supplement: S2 Fig — (TIF) [file pone.0208922.s002.tif]
